# Supplementary material for: Digitally engaged physicians about the digital health transition
Source: PLoS One. 2020 Sep 28;15(9):e0238658. doi: 10.1371/journal.pone.0238658 (PMC7521720; doi:10.1371/journal.pone.0238658)
Supplement: S1 File — (DOCX) [file pone.0238658.s001.docx]

**Supplementary Materials**

1. **Introduction**

Why did you choose to become a doctor?

What do you like most about your current work?

What do you like least about your current work?

What significant changes have you experienced in your profession / at work since you’ve been on your career?

How are you affected by these changes? How do you feel about them?

1. **Digital health associations**

What comes to mind when you hear the term “digital health”?

What impact does digital health have on your work?

**3. The 21st Century Physician**

**3.1. Technological changes / Current use**

Do you use digital health devices / services? If yes, what?

Do you encourage your patients to use such technologies? If so, in what way?

Which technologies would you like to use in your practice?

What do you think would be needed to incorporate these technologies into your practice?

What do you think the potential benefits of digital health can be?

What requirements must be met by digital health tools?

Do you feel that it is more difficult to separate yourself from your work?

What is the impact of the development of digital health on your private life?

What do you think the impact of digital technologies/health is on physicians’ stress, burnout and satisfaction?

**3.2. Physician-patient relationship**

How do you think digital health technologies affect your doctor-patient meetings/communication/decision-making? Do you have specific examples?

What benefits do you see in this change?

What are the most significant drawbacks?

How does digital health affect patient satisfaction/convenience/adherence?

How do you imagine the ideal doctor-patient relationship?

1. **Digital health and the future**

How do you see yourself and your role as a physician in five to ten years? How do you think your medical work will change in the next 5 to 10 years?

How do you imagine the average patient of the future?

What will an average doctor-patient visit/relationship look like in ten years?

What kind of changes do you think doctors are expected to make about digital health?

What are the implications of these changes for the skills physicians need?

How do you think medical training (e.g. university curriculum) should be changed regarding the use of new tools and other changes?
